# Performance of Sorghum Varieties under Variable Rainfall in Central Tanzania

**DOI:** 10.1155/2017/2506946

**Published:** 2017-04-27

**Authors:** Barnabas M. Msongaleli, S. D. Tumbo, N. I. Kihupi, Filbert B. Rwehumbiza

**Affiliations:** ^1^Department of Geography and Environmental Studies, University of Dodoma, P.O. Box 395, Dodoma, Tanzania; ^2^Department of Engineering Sciences and Technology, Sokoine University of Agriculture, P.O. Box 3003, Morogoro, Tanzania; ^3^Department of Soil and Geological Sciences, Sokoine University of Agriculture, P.O. Box 3008, Morogoro, Tanzania

## Abstract

Rainfall variability has a significant impact on crop production with manifestations in frequent crop failure in semiarid areas. This study used the parameterized APSIM crop model to investigate how rainfall variability may affect yields of improved sorghum varieties based on long-term historical rainfall and projected climate. Analyses of historical rainfall indicate a mix of nonsignificant and significant trends on the onset, cessation, and length of the growing season. The study confirmed that rainfall variability indeed affects yields of improved sorghum varieties. Further analyses of simulated sorghum yields based on seasonal rainfall distribution indicate the concurrence of lower grain yields with the 10-day dry spells during the cropping season. Simulation results for future sorghum response, however, show that impacts of rainfall variability on sorghum will be overridden by temperature increase. We conclude that, in the event where harms imposed by moisture stress in the study area are not abated, even improved sorghum varieties are likely to perform poorly.

## 1. Introduction

Sorghum (*Sorghum bicolor* L. Moench) is an important and widely adapted small-grain cereal grown in the tropics and subtropics and a staple food grain in food-insecure regions of Asia, Africa, and Central America [1]. Sorghum ranks second in importance after maize in Africa with a mean yield of 0.8 t/ha from a cultivated area of about 24 million hectares [2]. According to a review by Keya and Rubaihayo [3] sorghum ranks fifth after maize, cassava, rice, and wheat as staple in Tanzania. Nonetheless, sorghum plays a significant role in fighting hunger and food insecurity in central Tanzania.

Few long-term field experiments exist with sufficient detail in space and time to enable an understanding of variability in sorghum production due to dynamics in soil, nutrient, varieties, management, and weather processes and their interactions. Previous short-term field experiments at different locations and seasons, both on-farm and on-station, obtained higher grain yields, for instance, [4] (2.65 t ha^−1^) and [5] (2.58 t ha^−1^) for var. Tegemeo, contrary to the results by Saadan et al. [6] which showed that vars. Pato and Macia were superior to var. Tegemeo. Although short-term field experiments provide data with high degree of accuracy [7], they suffer from the failure to capture the interannual variability due to environmental conditions. Results from the previous experiments on improved sorghum varieties show that grain yields vary among varieties and across locations and seasons. Thus being short lived, the ensuing experiments do not permit derivation of robust conclusions about yield performance and adaptation of sorghum varieties over a long-term period.

Moreover, over the past years concerns have grown on increased rainfall variability across seasons resulting in large yield variability and thus becoming an apparent determinant on the performance and adaptation of sorghum varieties [8, 9]. Thus studies are essential which would combine long-term period and multiple locations (spatial-temporal analysis) under variable rainfall and soils to elucidate sorghum varieties' performance. Moreover, alongside such studies, analyses of rainfall trends are deemed necessary to understand the vulnerability of semiarid regions to historical and projected future conditions. Some rainfall analyses have shown decreasing trends (e.g., [10]) associated with decreases in the number of rainy days, while others have revealed neither abrupt changes nor trends [11]. These contrasting results suggest the need for undertaking location specific analyses of rainfall trends to ascertain contentious assertions on the same. A combination of field experiments and computer simulation models could be an appropriate option to comprehend the biophysical (climatic and soil conditions) factors and their interactions affecting crop yield and productivity [12, 13].

The Agricultural Production System sIMulator (APSIM) [14] is able to simulate growth and yield under different management practices and has been used by several studies under semiarid environments (e.g., [15, 16]). This study, therefore, used the APSIM model to simulate sorghum growth and yield patterns over the current (baseline) climate under existing soil conditions and local management practices across selected locations in semiarid central Tanzania. Specifically, the study used APSIM crop simulation model to investigate how rainfall variability may affect yields of improved sorghum varieties based on long-term historical rainfall and projected climate.

## 2. Materials and Methods

### 2.1. Study Area

The central zone comprising Dodoma and Singida regions is located between latitudes 6° and 06°08 S and longitudes 34°30′ and 35°45′ E. The experimental site was located at Hombolo Agricultural Research Institute (ARI) in Dodoma Region about 58 km North-East of Dodoma Municipality at latitude 05°45′ S and longitude 35°57′ E. The mean annual rainfall is 589 mm but the distribution is highly variable. The average annual temperature is 22.7°C. Soils at the experimental site are mainly sandy and loamy of low fertility. They are classified as Ferralic Cambisols in the FAO classification [17].

### 2.2. Experimental Design and Data Collection

Field experiments were conducted during 2012/13 and 2013/14 seasons. Three sorghum varieties, namely, Tegemeo, Macia, and Pato (the most widely grown varieties in the central zone), were used as treatments in a randomized complete block design (RCBD) with three replications. The recommended agronomic practices such as plant spacing and weeding are similar for the three varieties. Sowing was conditioned upon the previous day having received significant rainfall so as to wet the soil. Sorghum was sown at a spacing of 0.75 m between rows and 0.30 m within the row resulting in a plant density of 12 plants m^−2^. Weeding was done manually three times during the season on each plot using a hand hoe.

In order to provide near-optimum conditions, diammonium phosphate (DAP) fertilizer was applied during planting to supply 25 kg P/ha and 40 kg N/ha. Another round of N fertilization was done by applying 40 kg N/ha as Urea seven weeks after planting. The phenological data collected for the three sorghum varieties included date of flowering and date of physiological maturity. These were noted when 50% of plant population in each plot had attained that respective stage. Grain maturity was regarded to have been reached when dark spots at the point of attachment of the grain to the panicle started to show which was towards the end of April for both seasons. At final harvest, total aboveground biomass and grain yield were determined.

### 2.3. Historical Climatic Trends

Daily weather data during both seasons were obtained from observations at an agromet station, located about 500 m from the experimental plots. Past climate data (1961–2010) for selected weather stations, except Hombolo (1974–2010) in the central zone Tanzania, were analysed for trends. INSTAT plus (v3.36) software [18] was used to summarize the daily data into annual, monthly, and seasonal totals and to determine the onset taken as the first occasion after the earliest possible date on which a running total of at least 20 mm of rain was reached in four consecutive days with at least two days being wet and that no dry spell of 10 days or more occurred in the next 30 days [19]. Cessation of the rainy season was obtained through a water balance method and verified by visual daily display in INSTAT and length of growing period (LGP) was taken as the duration between the onset and cessation dates.

The Mann-Kendall test was used to test for significance of time series trends in total annual rainfall, seasonal rainfall, onset date, cessation date, and LGP. The Mann-Kendall test is less sensitive to outliers and has the capability to detect both linear and nonlinear trends and has been used in related studies in sub-Saharan Africa [20, 21]. The median measure was used to show onset and cessation dates and days of LGP as it is relatively unaffected by extreme values compared to the mean.

The Mann-Kendall test statistic is given as

(1) $$\begin{array}{c} S=\overset{N-1}{\underset{i=1}{\sum }}\;\overset{N}{\underset{j=i+1}{\sum }}\mathrm{sgn}\left(\;x_{j}-x_{i}\;\right), \end{array}$$

where *S* is the Mann-Kendall test statistic; *x*_*i*_ and *x*_*j*_ are the sequential data values of the time series in the years *i* and *j*  (*j* > *i*), and *N* is the length of the time series. A positive *S* value indicates an increasing trend and a negative value indicates a decreasing trend in the data series. The sign function is given as

(2) $$\begin{array}{c} \mathrm{sgn}\left(\;x_{j}-x_{i}\;\right)=\left\{\begin{array}{cc} +1 & \text{if }\left(x_{j}-x_{i}\right)>0\\ 0 & \text{if }\left(x_{j}-x_{i}\right)=0\\ -1 & \text{if }\left(x_{j}-x_{i}\right)<0. \end{array}\right. \end{array}$$

For *N* larger than 10, *Z*_MK_ approximates the standard normal distribution [22] and is computed as follows:

(3) $$\begin{array}{c} Z_{MK}=\left\{\begin{array}{cc} \frac{S-1}{\sqrt{Var\left(S\right)}} & \text{if }S>0\\ 0 & \text{if }S=0\\ \frac{S+1}{\sqrt{Var\left(S\right)}} & \text{if }S<0 \end{array}\right. \end{array}$$

The presence of a statistically significant trend is evaluated using the *Z*_MK_ value. In a two-sided test for trend, the null hypothesis *H*_0_ should be accepted if |*Z*_MK_| < *Z*_1−*α*/2_ at a given level of significance. *Z*_1−*α*/2_ is the critical value of *Z*_MK_ from the standard normal table (e.g., for 5% significance level, the value of *Z*_1−*α*/2_ is 1.96).

### 2.4. Model Description, Calibration, and Evaluation

The theory and parameterization of the APSIM model used in this study have been described in Ncube et al. [23]. APSIM has been tested in a diverse range of systems and environments, as well as model performance in long-term cropping systems in semiarid and subhumid environments in sub-Saharan Africa [24, 25]. The sorghum module used in the present study simulates the growth of a sorghum crop on a daily time step (on an area basis and not a single plant). Sorghum growth in this module responds to climate (temperature, rainfall, and radiation from the met module), soil water supply (from the SoilWat module), and soil nitrogen (from the SoilN module). Crop development is controlled by temperature (thermal degree days) and photoperiod. Thermal time accumulations were derived using an algorithm described in Jones and Kiniry [26] using observed phenology and weather data, a base temperature of 8°C, and an optimal temperature of 30°C. Genetic coefficients used by APSIM for sorghum are expressed in thermal degrees and photoperiod. The factor controlling the effect of photoperiod was set to a minimum value of 0.01 to eliminate the effect of photoperiod from the varieties as “modern” varieties are photoperiod insensitive [27]. In the present study, the APSIM model was evaluated for simulation of days after sowing to flowering and maturity, dry matter accumulation (biological yield), and grain yield.

Soil water dynamics between soil layers were defined by the cascading water balance method [28]. Its characteristics in the model are specified by the drained upper limit (DUL), lower limit of plant extractable water (LL15), and saturated water content (SAT). Soil characteristics of a soil profile opened up at the experimental site including soil texture, pH of soil, organic carbon content, and cation exchange capacity are shown in Table 1. Characteristics for the additional soil profiles used in simulations at different locations across the study area were obtained from the available soil databases (Table 2).

Each APSIM module demands a number of parameters. For the SOILWAT module, which simulates the dynamics of soil water, the inputs included soil bulk density, LL15 and DUL, and two parameters, *U* and CONA, which determine first- and second-stage soil evaporation. LL15 and DUL and SAT were estimated according to Saxton et al. [29]. The parameters, *U* and CONA, were set at 6.0 mm day 1 and 3 mm day 1, respectively, values acceptable for tropical conditions [30]. A value of 0.7 was used for SWCON, a coefficient that specifies the proportion of the water in excess of field capacity that drains to the next layer in one day [30]. The bare soil runoff curve number (cn2_bare) was set to 50 to account for the low runoff because of the flat topography and high infiltration rates due to the sandy soil nature of the experimental site ([31] cited by [32]). Parameters influencing soil fertility are mainly represented in APSIM-SoilN2 module. For the soil N model the organic carbon content for each soil layer was measured at the experimental site. The initial soil N was set at 25 kg/ha (20 kg NO_3_-N/ha and 5 kg NH_4_^+^-N/ha) for the top two layers based on published data around central Tanzania [33], and *P* was assumed nonlimiting.

The calibrated model was evaluated by comparing observed values for grain yield and total aboveground biomass with those from model simulations. Model performance was assessed through root mean square error (RMSE) [34],

(4) $$\begin{array}{c} \text{RMSE}=\sqrt{\frac{1}{N}}\sum {\left(\;\hat{Y}i-Yi\;\right)}^{2} \end{array}$$

and index of agreement or *d*-statistic [35],

(5) $$\begin{array}{c} d=1-\left[\;\frac{\sum_{i=1}^{n}\left(\hat{Y}i-Yi\right)\left(\hat{Y}i-Yi\right)}{\sum_{i=1}^{n}\left(\left|\hat{Y}i-\overset{-}{Y}i\right|+\left|Yi-\overset{-}{Y}i\right|\right)}\;\right], \end{array}$$

where $\hat{Y}$, *Y*, and $\overset{-}{Y}$ are, respectively, the simulated, observed, and mean of the observed values and *n* is the number of observations. For good agreement between model simulations and observations,* d*-statistic should approach unity.

### 2.5. Future Climate Data for Sorghum Yield Projections

Future climate data were obtained from Coupled Model Intercomparison Project phase 5 (CMIP5) under three Global Circulation Models (GCMs), namely, GFDL-ESM2M, HadGEM2-ES, and MIROC5 for mid-century RCP8.5 using the method by Hempel et al. [36]. Subsequently, simulations were performed for the three sorghum varieties under current (1980–2010) climate and yields compared with those obtained under future climatic conditions. The RCP8.5 is a high emissions scenario, corresponding to projections of high human population (12 billion by 2100), high rates of urbanization, and limited rates of technological change, all resulting in emissions approaching 30 Gt of carbon by 2100 compared with 8 Gt in 2000 [37].

### 2.6. Statistical Analysis

Analysis of variance (ANOVA) was used to analyse yield and total biomass data from the different treatments, with variety and replication, used as fixed and random effects, respectively. Test of significance between the 2012/2013 and 2013/2014 experiments was done using a *t*-test for pairwise comparison of means. Analysis of variance was performed using GENSTAT (v. 14) software (VSN international Ltd., Hempstead, England) whereas paired *t*-test was performed using Microsoft Excels' add-in Analyse-it (Analyse-it Software Ltd., The Tannery, 91 Kirkstall Road Leeds, LS31HS, United Kingdom).

## 3. Results and Discussion

### 3.1. Trends in Onset and Cessation Dates and Length of Growing Period

The median for onset of rainfall begins on the last week of November to first week of December (Table 3). Standard deviation varied between 11 and 15 days. The results indicated that the onset dates in the last 50 years have changed with all stations depicting early trends. However, the trends are not statistically significant except for Hombolo station. According to the analysed data, cessation of rainfall starts from the first week of April (at Hombolo) to last week of April (at Singida) (Table 3). Munishi [38] also reported similar findings in central Tanzania with slightly earlier onset and cessation dates. The median date for rainfall cessation was characterized by high standard deviation (>10 days) at all stations implying high variability in the pattern of end of the rainy season. However, these results are contrary to other studies which have shown less variable cessation dates than onset dates [19, 39].

Median LGP in the central Tanzania varied from 122 to 145 days depending on the location of the station (Table 3). All stations had higher coefficients of variation (>13%) in LGP which indicate high year to year variability of LGP except for Dodoma (12%). Higher coefficients of variation (>13%) in LGP give less confidence in crop selection based on maturity period. From the analyses, a mix of increasing and decreasing trends in LGP was obtained. Singida and Hombolo stations show statistically significant increasing trends in LGP (Table 3). However, Dodoma, Mpwapwa, and Manyoni stations had nonsignificant decreasing trends in LGP, results which are in agreement with findings from earlier studies which indicate that LGP has been shortening with a decreasing trend of number of rainy days during the growing season [19, 38, 40].

### 3.2. Climate Change Projections

Selected GCMs consistently projected increased temperatures for selected weather stations in the central zone of Tanzania. Projected temperature changes showed a mean increase in the range of 1.4–2.8°C (Table 4). In contrast, the projected change in rainfall across the stations showed decline, except for MIROC5, which showed an increase of +4.5–7.3% (Table 5). While projected rainfall changes were variable and uncertain, the projected temperature changes showed strong consistency with an upward trend.

### 3.3. Field Experimental Results

Table 5 shows grain yields and aboveground biomass obtained during the two experimental seasons.

There was no significant variation among varieties in the 2012/2013 season with respect to biomass at 50% anthesis, biomass at harvest maturity, and grain yield (Table 6). However, during the season of 2013/2014, significant variation (*P* < 0.05) in the three variables was observed among varieties. Further, there was interseasonal variation in plant biomass at 50% anthesis, grain yield,and biomass at harvest as indicated by the *t*-statistic in Table 6.

### 3.4. Model Calibration and Evaluation

Genetic coefficients used by APSIM for sorghum after calibration are shown in Table 7.

Comparison between observed and simulated grain and biomass yield combined for the two seasons is shown in Table 8. Statistical indicators show the simulation efficiency of APSIM model in simulating sorghum. Root mean square error (RMSE) which is an overall measure of model performance and compares simulated versus observed values shows a good agreement because the lower the values of RMSE the better the model in explaining most of the variations in the dataset. Moreover, data indicate that the simulated grain and biomass yield values reasonably matched observed values, owing to the agreement index (*d*-statistic) ranging from 0.6 to 0.9 across the varieties. The *d*-statistic values close to 1 are regarded as better simulations and according to these statistical indicators the model performance was deemed satisfactory to allow continuation of simulations both for long-term (temporal) and at different locations (spatial).

### 3.5. Influence of Water Stress on Sorghum Grain Yield

**Figure 1:**
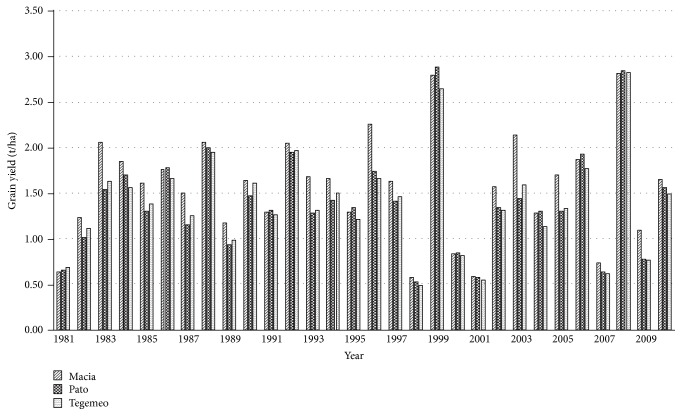
Simulated grain yield of sorghum varieties under baseline (1980–2010) conditions at Hombolo.

Simulated grain yields for the three varieties at the experimental station are shown in Figure 1. The simulation package consisted of planting between 15 December and 15 January, a row spacing of 0.90 m, and a population of 9 plants per m^2^ without N fertilizer under baseline weather (1980–2010). Results indicated that simulated yields varied among varieties with the range of 2.65–2.88 t ha^−1^ for the highest yields, and 0.48–0.57 t ha^−1^ for the lowest yields.

Further examination of rainfall and yields in 1998 (the year producing the lowest simulated yields) and 2008 (the year producing the highest simulated yields) demonstrates the importance of rainfall distribution during the growing period and especially during critical stages. There was approximately 0.50 t ha^−1^ maize yield in 1998 compared to 2.80 t ha^−1^ in 2008 (Figure 1). This was probably due to water stress. It means that yields simulated by APSIM are highly sensitive to wet/dry spell sequences during the crop growing season. Baigorria et al. [41] observe that not only is increasing persistence of wet/dry day occurrences important, but also the timing within the growing season is important when these wet/dry spells occurred. Decadal analyses of rainfall for occurrences of 5- and 10-day dry spells shown in Table 9 indicate that in 1998 the occurrence of a 10-day dry spell during the first decade in March caused strong water stresses which significantly reduced sorghum grain yields. On the contrary sorghum experienced only a brief water stress period (5-day dry spell during the same period); as a result much higher yields were obtained in 2008. According to the sowing dates in the simulation package, the period represents the crop growth stages from flag leaf appearance to start of grain filling. Premachandra et al. [42] indicate that as the most sensitive period for sorghum response to drought among phenological phases.

### 3.6. Simulations under Both the Baseline and Future Climates

**Figure 2:**
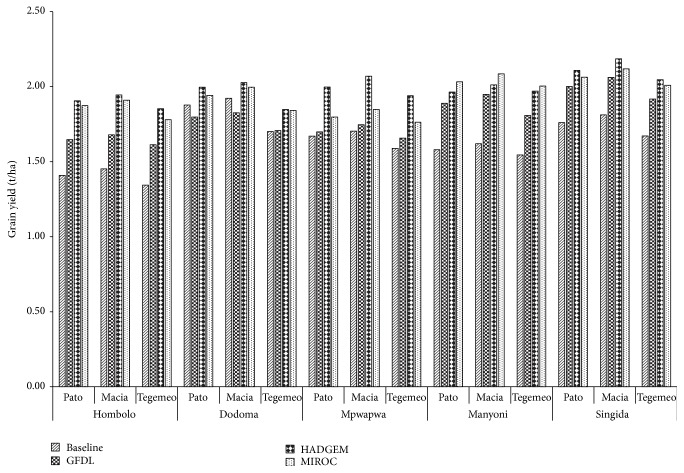
Simulated grain yields of Pato, Macia, and Tegemeo sorghum varieties under baseline and future climatic conditions in central Tanzania.

Mean simulated sorghum yields obtained from different locations (weather stations) across the central zone of Tanzania are shown in Figure 2. Taking into account uniform farmers' management practices across the study area, the simulated sorghum yields were envisaged to be influenced by the response to rainfall and soil variability. However, despite the differences in rainfall projections shown by the GCMs (Table 6), the simulated average sorghum yields were consistently higher under all GCMs (with HADGEM-ES giving the highest) compared with baseline for all the three sorghum varieties. The increased yield, therefore, may be attributed to temperature increases. Similarly, Turner and Rao and Zinyengere et al. [43, 44] find sorghum gaining in terms of grain yields from higher temperatures in specific regions with lower baseline temperatures (below 20°C). Simulation results from the current study could answer the questions about future development trajectory in the study area. Moreover, as discussed by Enfors et al. [45], increasing investments in small-scale water system technologies provides opportunities for the small-scale farming systems that dominate the study area to leverage the uncertainty of the future climates.

## 4. Conclusions

The field experimental results for the two seasons show considerable variations in grain yields among varieties. An early maturing variety Macia gave higher yields in both seasons compared to vars. Pato and Tegemeo. Model simulated yields reveal that the length and timing of dry spells during the growing season are major determinants of grain yields and they surpass total seasonal rainfall amount even for a hardy crop like sorghum. Results suggest that occurrence of a long dry spell (10-day or longer) during the period from flag leaf appearance to start of grain filling is critical and could significantly reduce yield. Therefore, considering the inability of smallholder farmers to construct and maintain rain-water harvesting (RWH) structures, the government and development partners should consider increasing investments in the same to ensure supplemental irrigation during critical stages. The availability of water would enable smallholders growing sorghum to leverage the uncertainty in climate, but also to tap the opportunities brought in by increased temperatures. The phenological characterization of the three varieties and subsequent calibration and validation of APSIM have provided a basis on which various kinds of simulations could be done with the aim of increasing and sustaining sorghum productivity.

## Figures and Tables

**Table 1:** Soil physical and chemical properties used for the calibration of APSIM.

Soil parameters	Layers					
	150 mm^a^	150 mm^a^	150 mm^a^	250 mm^a^	350 mm^a^	300 mm^a^
BD (g cm^−3^)	1.38	1.47	1.44	1.38	1.51	1.51
SAT (cm cm^−1^)	0.37	0.35	0.34	0.33	0.33	0.33
LL (cm cm^−1^)	0.084	0.084	0.134	0.134	0.134	0.14
DUL (cm cm^−1^)	0.248	0.299	0.334	0.278	0.270	0.270
Clay (%)	19	20	23	25	34	30
Silt (%)	5	4	4	5	2	4
CEC (cmol/kg)	6.0	8.2	9.2	10.2	10.0	6.0
Soil C parameters						
Organic C (g 100 g^−1^)	0.41	0.31	0.23	0.14	0.14	0.06
Finert^b^	0.4	0.6	0.8	0.8	0.9	0.9
Fbiom^c^	0.025	0.02	0.015	0.01	0.01	0.01

BD: bulk density; SAT: volumetric water content at saturation. LL is wilting point (volumetric water content at −15 bar pressure potential) and DUL is drained upper limit.

^a^Layer thickness (mm).

^b^Proportion of soil carbon assumed not to decompose.

^c^Proportion of decomposable soil carbon in the more labile soil organic matter pool.

**Table 2:** Soil properties of the profiles used in simulations across stations.

Properties	Dodoma	Hombolo	Mpwapwa	Manyoni	Singida
Soil layers/depth (cm)	6/135	6/135	4/110	4/115	4/110
Sand, silt, clay (% in 0–15 cm)	79, 5, 16	79, 5, 16	81, 6, 13	66, 10, 14	55, 21, 24
Textural class	Sandy loam	Sandy loam	Sandy loam	Sandy loam	Sandy clay loam
Plant available water	119.2	119.2	112.8	164.1	162.1
Organic carbon (top three layers)	0.32, 0.21, 0.11	0.32, 0.21, 0.11	0.45, 0.30, 0.15	0.56, 0.32, 0.12	0.52, 0.38, 0.20

Source: AfSIS.

**Table 3:** Statistical characteristics and trends of onset date, cessation date, and LGP at five stations over the period 1961–2010 in central Tanzania.

Station	Statistics	Dodoma	Mpwapwa	Hombolo	Manyoni	Singida
Onset	Median	Dec 13	Dec 7	Dec 7	Dec 1	Nov 26
	*Z*_MK_	−0.07^ns^	−0.030^ns^	−1.196^*∗*^	−0.911^ns^	−0.680^ns^
	Slope	0.00	−0.091	−0.321	−0.225	−0.131
	SD	11.311	14.252	14.870	14.361	14.582

Cessation	Median	Apr 18	Apr 13	Apr 5	Apr 14	Apr 30
	*Z*_MK_	−0.337^ns^	−1.188^ns^	0.970^ns^	−0.755^ns^	1.692^*∗*^
	Slope	0.000	−0.083	0.029	−0.070	0.303
	SD	10.252	16.041	11.054	14.281	16.711

LGP (days)	Median	124	122	123	141	145
	*Z*_MK_	−0.303^ns^	−0.419^ns^	2.092^*∗*^	−0.480^ns^	1.876^*∗*^
	Slope	0.000	−0.067	0.434	0.000	0.692
	CV (%)	12.510	13.711	14.281	13.511	15.982

*Z*_MK_ is Mann-Kendall trend test, slope (Sen's slope) is the change (days)/annum; *∗* is statistically significant at 0.05 probability level; ns is nonsignificant trend; SD is standard deviation; CV is coefficient of variation.

**Table 4:** Mean change in projected climate between baseline (1980–2010) and mid-century (2040–2069) RCP8.5.

Station	GCM	Temperature (°C)			Rainfall (%)
		Average	Minimum	Maximum	
Dodoma	GFDL-ESM2M	1.4	1.7	1.2	−8.5
	HADGEM2-ES	2.8	2.9	2.8	−1.4
	MIROC5	2.2	2.1	2.4	7.3

Manyoni	GFDL-ESM2M	1.8	1.8	1.7	−3.0
	HADGEM2-ES	2.7	2.6	2.8	−5.2
	MIROC5	2.3	2.1	2.4	7.0

Singida	GFDL-ESM2M	1.8	1.8	1.7	−1.9
	HADGEM2-ES	2.7	2.6	2.8	−2.7
	MIROC5	2.3	2.1	2.4	4.5

**Table 5:** Grain yield, aboveground biomass, and harvest index for seasons 2012/13 and 2013/14.

Variety	2012/13		2013/14		Combined seasons		
	Grain yield (kg/ha)	Aboveground biomass (kg/ha)	Grain yield (kg/ha)	Aboveground biomass (kg/ha)	Days to 50% flowering	Days to harvest maturity	Plant height (max) mm
Macia	4064^*∗*^	10517	4355	11388	65	102	1290
Pato	3896	11411	4088	12394	76	118	1780
Tegemeo	3798	10843	4012	11415	74	114	1650
S.E.D	233.9	274.3	79.1	100.7	0.577	0.471	147.1

^*∗*^Means over three replications. S.E.D = standard error of differences of means.

**Table 6:** Intra- and interseasonal variation in biomass, grain yield, and tops weight.

Variable	2012/2013	2013/2014	*t*-statistic
Biomass at 50% anthesis	2.77^ns^	5.49^*∗*^	3.89^*∗∗*^
Grain yield at harvest	1.41^ns^	21.08^*∗*^	5.08^*∗*^
Biomass at harvest maturity	3.23^ns^	50.49^*∗*^	8.60^*∗*^

^*∗*^Significant at *P* < 0.05; ^*∗∗*^significant at *P* < 0.01; ns = not significant.

**Table 7:** Crop parameters for three sorghum cultivars used for the simulations in APSIM.

Parameter		Source	Units	Macia	Tegemeo	Pato
Thermal time accumulation	End of juvenile phase to panicle initiation	C	°C day	230	270	275
	Flag stage to flowering	C	°C day	195	170	175
	Flowering to start of grain filling	C	°C day	80	80	100
	Flowering to maturity	C	°C day	675	760	760
	Maturity to seed ripening	L	°C day	1	1	1
Photoperiod	Day length photoperiod to inhibit flowering	D	H	11.5	11.5	11.5
	Day length photoperiod for insensitivity	D	H	13.5	13.5	13.5
	Photoperiod slope	L	°C/h	0.01	0.01	0.01
	Base temperature	L	°C day	8	8	8
	Optimum temperature	D	°C day	30	30	30
	Plant height (max)	O	mm	1290	1650	1780

C: calibrated; D: default; L: literature; O: observed.

**Table 8:** Statistical indicators of model performance.

Parameters/cultivar	Macia		Tegemeo		Pato	
	RMSE (kg/ha)	*d*-Stat	RMSE (kg/ha)	*d*-Stat	RMSE (kg/ha)	*d*-Stat
Grain yield	133	0.73	87	0.62	140	0.60
Biomass	178	0. 93	418	0.66	236	0.83

**Table 9:** Occurrences of dry spells during March and April and their relationship to simulated grain yields at Hombolo.

	Years with the lowest yields												Years with the highest yields											
	1998						2001						1999						2008					
	MAR			APR			MAR			APR			MAR			APR			MAR			APR		
DEKAD	1	2	3	1	2	3	1	2	3	1	2	3	1	2	3	1	2	3	1	2	3	1	2	3
5-day		**√**						**√**	**√**	**√**		**√**			**√**	**√**	**√**	**√**	**√**	**√**				
10-day		**√**						**√**				**√**						**√**						
Yield (t/ha)	0.48–0.57						0.54–0.58						2.65–2.88						2.82–2.84					
Rain (mm)	38.8			98.9			66.4			63.2			211.6			117			182.1			42.3		
Rainy days	6			8			6			8			12			5			10			11		

√ indicates occurrence of a dry spell in a decade (10-day interval) within a month.
